# Genotyping of black grouse MHC class II B using reference Strand-Mediated Conformational Analysis (RSCA)

**DOI:** 10.1186/1756-0500-4-183

**Published:** 2011-06-14

**Authors:** Tanja M Strand, Jacob Höglund

**Affiliations:** 1 Population Biology and Conservation Biology, Dept. of Ecology & Genetics, Evolutionary Biology Centre, Uppsala University, Norbyvägen 18D, SE-752 36, Uppsala, Sweden

## Abstract

**Background:**

The Major Histocompatibility Complex (MHC) is a cluster of genes involved in the vertebrate immune system and includes loci with an extraordinary number of alleles. Due to the complex evolution of MHC genes, alleles from different loci within the same MHC class can be very similar and therefore difficult to assign to separate loci. Consequently, single locus amplification of MHC genes is hard to carry out in species with recently duplicated genes in the same MHC class, and multiple MHC loci have to be genotyped simultaneously. Since amplified alleles have the same length, accurate genotyping is difficult. Reference Strand-Mediated Conformational Analysis (RSCA), which is increasingly used in studies of natural populations with multiple MHC genes, is a genotyping method capable to provide high resolution and accuracy in such cases.

**Findings:**

We adapted the RSCA method to genotype multiple MHC class II B (BLB) genes in black grouse (*Tetrao tetrix*), a non-model galliform bird species, using a 96-Capillary Array Electrophoresis, the MegaBACE™ 1000 DNA Analysing System (GE Healthcare). In this study we used fluorescently labelled reference strands from both black grouse and hazel grouse and observed good agreement between RSCA and cloning/sequencing since 71 alleles were observed by cloning/sequencing and 76 alleles by RSCA among the 24 individuals included in the comparison. At the individual level however, there was a trend towards more alleles scored with RSCA (1-6 per individual) than cloning/sequencing (1-4 per individual). In 63% of the pair-wise comparison, the identical allele was scored in RSCA as in cloning/sequencing. Nine out of 24 individuals had the same number of alleles in RSCA as in cloning/sequencing. Our RSCA protocol allows a faster RSCA genotyping than presented in many other RSCA studies.

**Conclusions:**

In this study, we have developed the RSCA typing method further to work on a 96-Capillary Array Electrophoresis (MegaBACE™ 1000). Our RSCA protocol can be applied to fast and reliable screening of MHC class II B diversity of black grouse populations. This will facilitate future large-scale population studies of black grouse and other galliformes species with multiple inseparable MHC loci.

## Background

In vertebrates, MHC (Major Histocompatibility Complex) is a cluster of highly variable genes that plays an important role in the immune defence and which may evolve through different forms of balancing selection [1-4]. This multigene family is highly interesting from an evolutionary perspective. For example, MHC genes have been associated with individual fitness, population viability and evolutionary potential in changing environments [2]. MHC genes are in fact among the most suitable candidates for studies of adaptive genetic diversity because of the numerous associations found between MHC genotypes and pathogen resistance e.g. [5-9]. In studies of both model and non-model species, the interest in MHC genes has remained high among evolutionary biologists over decades. However, large-scale studies of natural populations are hampered because of the complexity working with MHC genes.

MHC evolution is characterized by repeated gene duplication (birth) and gene loss (death), thereby loci evolve under a birth-and-death model [10]. Supplementary to the birth-and-death model of MHC evolution is concerted evolution, observed particularly in birds [11,12]. This is the result of frequent genetic exchange between duplicated genes by recombination or gene conversion (i.e. interlocus genetic exchange). Because of the complicated (and not fully understood) evolution of MHC genes there are pronounced differences in the genomic organization and number of the MHC loci between vertebrate lineages [13], especially between mammalian and non-mammalian species [12,14]. For this reason it is a great challenge to accurately amplify MHC genes in non-model species. For example, the number of MHC loci can vary extensively between different species, even when the species compared are phylogenetically related and belong to the same taxonomic family, like chicken and quail [15]. There are also known or assumed cases of MHC copy number variation within species [16-21]. Additionally, in non-mammalian species with multiple loci per MHC class, the loci belonging to the same MHC class are, in most species, too similar to tell apart [16,22-25]. Designing primers that amplify a single MHC locus is therefore a difficult task. In combination with PCR artefacts [26] and the common presence of pseudogenes in MHC, this also makes single locus amplification of functional MHC alleles hard to accomplish. Consequently, many MHC studies in non-mammalian species are struggling with genotyping PCR products including more than two MHC alleles.

For PCR products from multiple MHC loci, existing genotyping methods, like cloning and Sanger sequencing, Reference Strand-Mediated Conformational Analysis (RSCA), Single Strand Conformation Polymorphism (SSCP), Denaturing Gradient Gel Electrophoresis (DGGE) and Next Generation amplicon sequencing, work with different levels of accuracy, precision and speed [27-29]. RSCA, DGGE and SSCP all are methods that have the potential to separate PCR products that have the same length but differ in DNA sequence. PAGE-SSCP (gel-based SSCP) is a widely used MHC typing method that can separate alleles that only differ in one base-pair because of the different conformation of the alleles [30]. The method has earlier been modified for use on automated sequencers [31] and recently also for capillary electrophoresis, CE-SSCP [32]. In contrast, DGGE has not, to our knowledge, been adapted to automated sequencers [33]. This technique is based on the principle that double-stranded DNA molecules have unique denaturation rates that are based on the specific nucleotide composition.

**Figure 1:**
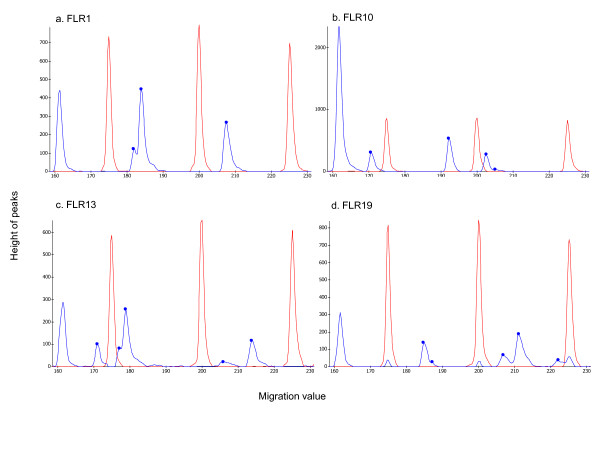
**RSCA electropherograms**. Examples of RSCA electropherograms from the MegaBace™ 1000 for one individual "Ref26" using all four FLRs, a) FLR-Tete1, b) FLR-Bobo1, c) FLR-Bobo2 and d) FLR-Bobo3. On the x-axis are the migration values in the non-denaturing gel, the y-axis reflects the height of the peaks. The red peaks are the size markers and the blue peaks are the RSCA-peaks. The left most of the blue peaks are the FLR-homoduplex peak. The other blue peaks are used in the RSCA-scoring.

RSCA [34] is a genotyping method increasingly used in studies of mammalian MHC but recently also in the Seychelles warbler [35], red jungle fowl [36], peacock [37], stickleback [28] and lake trout [38]. In RSCA, a fluorescently labelled reference DNA fragment is generated by PCR and hybridised with the PCR products of the samples to be tested ("test alleles"). A duplex is formed containing loops and bulges corresponding to the number and position of base pair mismatches between the fluorescent labelled reference strand (FLR) and each test strand [34]. When electrophoresis is run through a non-denaturing polyacrylamide gel, each different duplex formed will migrate slightly differently. In a laser detection system, the relative positions of the fluorescent duplexes are shown as different peaks in an electropherogram, two peaks per heterozygote individual and a single peak for a homozygote if amplifying a single locus. Each allele will have a specific migration value (peak) in the electropherogram when run with a specific FLR, and running each allele with several different FLRs enables differentiation between highly similar alleles. Each individual is therefore RSCA genotyped with a number of different FLRs. For examples of RSCA electropherograms, see Figure 1. To be able to identify known or new alleles from the peaks (migration values), a RSCA library is first constructed with the migration values from each known allele (see below). The superior advantage of RSCA compared to SSCP and DGGE is the usage of multiple FLRs that minimizes problems with co-migration of alleles.

Although time-consuming, RSCA methods where preparation of acrylamide gels are required, is still widely used for MHC genotyping such as ALFexpress [39-43] and on ABI377 [44-46]. RSCA has also been optimised to various Capillary Electrophoresis (CE) instruments, firstly to the one capillary instrument ABI PRISM 310 (Applied Biosystems) [47,48]. Recently, besides CE instruments, also Capillary Array Electrophoresis (CAE) instruments, with several capillaries for faster genotyping, are used in RSCA typing, four capillaries in CAE ABI 3130 [28,49] and 16 capillaries in CAE ABI 3100 [21,28,36,50,51]. To our knowledge, RSCA genotyping has not previously been optimised for a MegaBACE™ instrument (GE Healthcare). RSCA on MegaBACE™ may work differently than RSCA on ABI because of the many differences between the systems. The dissimilarities between MegaBACE™ and ABI includes "the mechanism by which sample DNA is introduced into the capillaries, the capillaries themselves, i.e., capillary diameter, length, and internal coating, the mechanism of excitation (although both use laser-induced fluorescence), where the DNA is detected and the method of detection itself" [52].

The black grouse (*Tetrao tetrix*) has a well-studied complex mating system where males defend territories at traditional leks (mating grounds) which females visit to mate (Höglund and Alatalo 1995). Also, the black grouse is a wide-spread bird with both viable and threatened populations and MHC studies can, for example, clarify the role and importance of MHC diversity in conservation. In an earlier study [25] we amplified and sequenced transcribed MHC class II B alleles in black grouse, but before we can continue with large-scale studies of MHC variation, we wanted to design an efficient and reliable MHC genotyping method suitable for black grouse.

The objective of this study was to develop an RSCA approach further, for even higher throughput MHC genotyping on a 96-Capillary Array Electrophoresis (the MegaBACE™ 1000, an instrument not previously used for RSCA) in general, and more specifically, for MHC genotyping in black grouse. Such a method could facilitate large-scale surveys of MHC genetic polymorphism in both model and non-model species. To verify the method we compared the RSCA typing results with cloning and classic Sanger sequencing. In our study, we successfully genotyped black grouse MHC class II B alleles from expressed loci using RSCA. When we compared cloning/sequencing with RSCA, most black grouse alleles were detected with both methods. There is however a tendency for higher number of MHC alleles typed with RSCA than cloning/sequencing. We therefore suggest that our RSCA genotyping protocol is not primarily suitable for individual level studies of MHC diversity where allele-specific correlations are sought for, but it is appropriate for large-scale studies of MHC diversity on the population level.

## Results

### Amplification of MHC BLB

The primer pair RNAF1a (5'GACAGCGAAGTGGGGAAATA-3') and RNAR1a (5'- CGCTCCTCTGCACCGTGA-3') was used to amplify a 125 bp fragment of the BLB exon 2 in all following PCR protocols. We designed these primers in conserved regions of the exon 2 for our previous study and have shown that they specifically amplify functional BLB but not YLB alleles [25]. We assume that this primer pair is capable of amplifying a wide range of different BLB alleles, as it amplifies the BLB as well in two related grouse species, the hazel grouse and the willow grouse (*Bonasa bonasia *and *Lagopus lagopus*, unpublished data).

### RSCA establishment

#### FLR selection

Ideally, the alleles chosen to be FLRs should neither be too different nor too similar to the alleles genotyped ("test alleles") in the study [50]. If the FLR and the test allele are too different, they may not hybridise. On the other hand, if the FLR and the test allele are too similar, the homoduplex formation of the FLR and the heteroduplex formation between the FLR strand and the test allele strand will co-migrate in the gel and the test allele will therefore become undetected. Co-migration can also happen when a FLR strand is hybridised with a test allele strand, if the hybridisation produces a formation that has similar migration speed as another test allele. One way to avoid co-migration and therefore avoid undetected alleles is to use more than one FLR. The risk of co-migration with the homoduplex is also decreased if the FLRs are chosen from a related species instead of the same species, since shared alleles between different species are presumably rare.

In this study, we used one allele from black grouse and three alleles from a species from the same subfamily (Tetraoninae), the hazel grouse [53], giving a total of four FLRs. The first allele cloned and sequenced from our previous study [25], *Tete BLB01*, was assigned FLR-Tete1. We tested the remaining black grouse alleles, *Tete BLB02-09*, as FLRs and most of them resulted in distinguishable peaks in the electropherogram. However, we decided to use FLRs from the hazel grouse, to reduce the risk of undetected test-alleles identical to the allele that the FLR is based on. Using hazel grouse alleles minimizes this risk since black grouse and hazel grouse probably do not share alleles (although possible due to trans-species polymorphism). MHC class II B alleles were amplified from one hazel grouse individual (cloned twice) using the same primers (RNA F 1a and RNA R 1a), PCR-protocol and master cycler program as for the black grouse BLB (see [25]). The PCR-product was ligated into pCR4-TOPO vector using a Topo TA cloning kit (Invitrogen, Carlsbad, CA, USA) following the protocol by the manufacturer. Twenty colonies were both forward and reverse sequenced on MegaBACE™ 1000 DNA Analysis System (GE Healthcare, Uppsala, Sweden). The resulting three unique hazel grouse sequences were all chosen for FLRs. Allele *Bobo BLB01 *was assigned FLR-Bobo1, *Bobo BLB02 *FLR-Bobo2 and *Bobo BLB03 *FLR-Bobo3. GenBank accession numbers are GQ851943 for *Bobo BLB01*, GQ851944 for *Bobo BLB02 *and GQ851945 for *Bobo BLB03*.

#### FLR amplification

We constructed a FAM-fluorescently labelled forward primer FAM RNAF1a and used the normal non-labelled reverse-primer RNAR1a for the FLR PCR on the above clones for FLR-Tete1, FLR-Bobo1, FLR-Bobo2 and FLR-Bobo3. We used ten times as much of the labelled primer than the non-labelled one, to increase the heights of the heteroduplex peaks in the electropherogram [21]. Approximately 50 ng DNA, 0.5 μM of the FAM forward primer, 0.05 μM of the reverse primer, 0.6mM of dNTP, 3 mM MgCl2, 1 x buffer and 0.75 units BioTaq polymerase (DNA Technology, Aarhus, Denmark) were used in the 100 μl FLR PCR reaction. The reaction was initiated at 94°C for 1 minute and was then run for 35 cycles at 94ºC for 1 minute and 30 seconds at both 64.9ºC and 72ºC before a stop for 10 minutes on 72°C. The PCR products were purified with Montage PCR spin columns (Millipore Cooperation, MA, USA), dissolved in TE buffer of pH 7 and stored at -20°C.

#### Construction of RSCA library

We first constructed a preliminary RSCA library with eight unique *BLB *sequences from our previous study [25]. The following PCR was performed on these eight clones. Approximately 50 ng DNA, 0.48 μM of each primer, 0.6mM of dNTP, 3 mM MgCl_2_, 1 x buffer and 0.75 units BioTaq polymerase (DNA Technology, Aarhus, Denmark) were used in a 25 μl PCR reaction. The reaction was initiated at 94°C for 1 minute and was then run for 30 cycles at 94ºC for 1 minute and 30 seconds at both 64.9ºC and 72ºC before a stop for 10 minutes on 72°C. The four FLRs were run with each of these original eight unique sequences (i.e. clones representing alleles *Tete BLB01-BLB09 *but not *BLB08 *since that allele may be an artefact) between four and nine times (mean 6.2 times) in independent runs, using different non-denatured polymer lots. Standard deviations (SD) for migrations values were calculated for each FLR using several runs. Between RSCA runs, the MegaBACE™ was run with denaturing polymer for microsatellite genotyping or sequencing using the same capillaries. The homoduplex FLR peak always had a migration value of about 161 bp, close to the size of the amplified part of exon 2 including primers. All RSCA peaks turned up to the right of the FLR peak (Figure 1). When we started to screen MHC alleles in a population study of black grouse (Strand et al. manuscript) using this RSCA library, we noticed new RSCA peaks so we cloned more individuals and increased the library with eight additional unique MHC class II B sequences. This updated RSCA library is based on 16 cloned MHC class II B sequences/alleles in total (additional file 1: RSCA library). One RSCA peak is visible for each unique allele in both FLR-Tete1 and FLR-Bobo1. FLR-Bobo2 shows two peaks (one additional or one alternative peak) instead of one peak for five alleles, and three peaks for one allele. One allele run with FLR-Bobo3 also shows an extra peak.

### RSCA genotyping

To compare RSCA typing with cloning and Sanger sequencing, we typed and scored the RSCA for individuals previously cloned in Strand et al. 2007 [25] and Strand et al. manuscript, using the RSCA library (additional file 1: RSCA library). After genotyping RSCA in MegaBACE™ 1000, we viewed our electropherograms in the software MegaBACE Fragment Profiler™ version 1.2 (GE Healthcare) and assigned possible alleles (see Figure 1 and Table 1 for an example) for each FLR using the RSCA library and scoring rules.

**Table 1:** RSCA migration values

	FLR-Tete1		FLR-Bobo1		FLR-Bobo2		FLR-Bobo3	
**Ind.**	**migr. value**	**alleles**	**migr. value**	**alleles**	**migr. value**	**alleles**	**migr. value**	**alleles**

Ref26	181.9	7,9,	170.6	2,7,16,	171	1,3,4,18,	184.8	5,
	184	7,9,22,	192.2	5,11,21,23,	177.1	9,11,	*187.2*	5,
	207.6	5,6,	202.6	1,	178.8	9,22,16,	206.9	16,22,
			*205.1*	1,3,22,	*205.9*	5',21,18'	211.2	2,9,22,
					213.8	5,6'	222.1	1,

An example of basic data for an individual (Ref26) after RSCA genotyping, before implementing the scoring rules. For each FLR, the (peak) migration values from the electropherogram and the corresponding possible MHC alleles having that migration range are reported. The possible MHC alleles should be read "*Tete BLB*" before the number. Numbers in italics are weak peaks in the electropherogram. Alleles with an apostrophe (') reflect a second or alternative RSCA peak.

The scoring rules were performed as follows:

1) A peak was scored as a certain allele if it were present in the migration value range of that allele (additional file 1: RSCA library) in four out of the four FLRs.

2) It is enough to score an allele as present if it exists in three FLRs if that allele is single in one strong peak (this means that no other allele has the same migration value in that FLR, for an example see allele 5 in migration value 184.8 in FLR-Bobo3, Table 1).

Exceptions to these rules exist for allele *BLB01 *since this allele is the basis for FLR-Tete1 (they are identical). FLR-Tete1 can therefore not have a peak for *BLB01*. In case *BLB01*, three FLRs is therefore enough for scoring in the first rule, and two FLRs is enough for scoring in the second rule.

Individuals present in the comparison between RSCA and cloning (Table 2) must first pass the two following criterions. First, at least 20 randomly chosen clones must have been picked per individual PCR. We sequenced 16-35 clones per individual (i.e. clones that had inserts of the right size, Table 2), Second, the reported MHC sequences must have been found in at least one other cloning of a different PCR reaction (in MHC studies, generally only alleles present in two independent PCR reactions are regarded as confirmed [27]).

**Table 2:** Comparison between cloning and RSCA in 24 individuals

	Cloning			RSCA	
**Individual**	**N clones**	**Alleles by seq**	**N alleles**	**RSCA peaks**	**N alleles**

D320	17	1,6,7,	3	1,6,7	3

H070	16	1,5,6	3	1,5,9,11,23	5

H070.3	23	1,3,6,9	4	1,3,6,9	4

H070.4	17	6,9	2	1,2,6,7,9	5

D375	16	1,5	2	1,5	2

H071	20	1,2,4,5	4	1,2,3,4,5,18,	6

H071.2	19	1,5	2	1,5	2

H071.3	20	1,2,4,5	4	1,4,5	3

H071.7	17	1,2,4,5	4	1,4,5	3

D476	17	1,3,5,9	4	1,2,3,5	4

D870	26	1,2,3,4	4	1,2,3,4,11	5

H904	16	1,5	2	1,5	2

D115804	16	1,4	2	1,4	2

H369	21	1,2,5	3	1,2,4,5	4

D249	20	6,11	2	1,2,6,11,23	5

H393	20	1,4,5,6,	4	4,5,6	3

Ref4	32	9,13,18	3	9	1

Ref24	28	22	1	1,9,22	3

Ref25	33	5,22	2	1,5,22	3

Ref26	27	1,5,22	3	1,5,22	3

Ref30	35	5,7,9,16	4	1,5,7,16	4

Ne36	17	1,22,	2	1	1

D248	33	4,6,7,9	4	1,9	2

10222	31	5,14,22	3	5	1

***Sum alleles***			***71***		***76***

***Mean alleles***			***2.96***		***3.17***

The two methods of cloning/sequencing and RSCA for scoring MHC class II B in black were compared. The table shows the different alleles "Alleles by seq" and number of alleles per individual "N alleles". The numbers below the columns named "Alleles by seq" refers to confirmed *Tete BLB *alleles. "N clones" refers to the number of clones sequenced per individual. Black grouse named "Ref" where sampled in England, "Ne36" from Netherlands, and had their DNA extracted from moulted feathers. Individual 10222 was sampled in Norway and the DNA was extracted from muscle. For the remaining (Finnish) samples, DNA was extracted from blood. 71 alleles were found by cloning and 76 by RSCA. 2.96 alleles (SD 0.95) were obtained in cloning, and 3.17 alleles (SD 1.40) in RSCA.

A typical example of scoring is presented for individual Ref 26. The electropherograms for Ref 26 for all four FLRs is visible in Figure 1. The leftmost blue peak at migration value of approx. 161 is the homoduplex peak for the FLR so that peak is always disregarded. One FLR show three RSCA peaks, one show four RSCA peaks and two show five RSCA peaks. Each RSCA peak lays in a certain migration value range for a specific MHC allele, as reported in Table 1.

In certain cases there are two or even three RSCA peaks in the same FLR for the same allele (probably because more than one formation is stable between the FLR and the allele), resulting in two (or three) migration value ranges within the same FLR for one allele (additional file 1: RSCA library). In the example in Table 1, alleles with an apostrophe (') reflect a second or alternative RSCA peak. Following the scoring rules (in the paragraph above) on Table 1, Ref 26 has allele *BLB22 *according to rule 1, allele *BLB05 *according to both rule 1 and rule 2, and *BLB01 *according to rule 2 (and rule 1 applying the exception for *BLB01*). This individual thus has three MHC alleles, namely *BLB01*, *BLB05 *and *BLB22 *according to RSCA. Cloning and sequencing also revealed these MHC alleles (28 clones sequenced). There are peaks present in the electropherogram (Figure 1, Table 1) that were not scored as alleles (181.9 (FLR-Tete1), 170.6 (FLR-Bobo1) and 177.1 (FLR-Bobo2)). Two of these peaks (181.9 (FLR-Tete1) and 177.1 (FLR-Bobo2)) are actually peaks within much larger peaks and could therefore be artefacts. The other peak (170.6 (FLR-Bobo1)) could represent an additional folding structure that may appear for example when alleles are hybridised with the FLR (because the sequences can match in more than one way) or due to either non-optimal hybridisation temperatures or MegaBACE run temperatures. This peak could simply also represent an unknown allele.

#### Comparison between cloning/sequencing and RSCA

**Figure 2:**
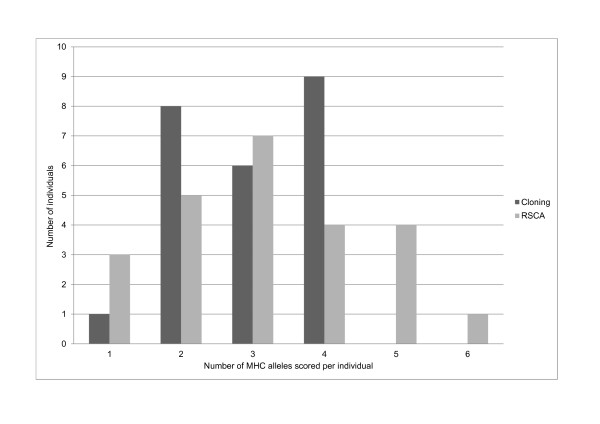
**Comparison cloning versus RSCA**. Number of MHC class II B alleles scored per individual obtained from cloning/sequencing versus RSCA genotyping. One to four alleles were visible in cloning and Sanger sequencing. One to six alleles were scored in RSCA.

The mean number of alleles typed per individual was 2.96 (SD 0.95) with cloning and 3.17 (SD 1.40) with RSCA among the 24 individuals but there were no significant difference in number of scored alleles (76 for RSCA, 71 for cloning, Table 2) (paired Wilcoxon signed rank test, df = 47.5, p = 0.487). Cloning and RSCA gave equivalent results in the majority of pair-wise comparisons for each allele scored with either of the two methods. With pair-wise we mean pairs between each allele (in all 24 individuals) observed in cloning and in RSCA, and we had 90 such pairs in our comparison (Table 2). In 57 out of 90 pair-wise comparisons, the same allele was scored in RSCA as in cloning. In 15 out of these 90 cases, an allele was observed in cloning but not in RSCA. Conversely, in 18 cases an allele was only observed in RSCA. The nucleotide sequence of *Tete BLB02 *deviates from *Tete BLB01 *with only one base pair (in the 163 base pair long fragment) but could still be distinguished in some individuals using RSCA. In three individuals, *Tete BLB02 *is only found using RSCA but not in cloning. In two other individuals, the same allele *Tete BLB02 *is found only in cloning. In three individuals, *Tete BLB02 *is found using both methods. Overall, comparing cloning and RSCA, discrepancies exists, but in both directions. However, RSCA may score more alleles than cloning (Figure 2). By cloning and sequencing, 1-4 alleles per individual are observed, and by RSCA, 1-6 alleles are scored per individual.

## Discussion

RSCA has been increasingly used as a MHC genotyping method in birds [35-37] and we have now further developed the RSCA typing method to operate on a 96-capillary array electrophoresis, providing a higher throughput. To our knowledge, this is the first time RSCA have been optimised to be run on the MegaBACE™ 1000 DNA Analysing System. We have now designed a genotyping method suitable for functional MHC class II B loci in black grouse. This method performs well on DNA extracted from both blood and moulted feathers. In the present study, when comparing cloning and classic Sanger sequencing with RSCA, most black grouse MHC class II B alleles are detected with both methods. Among the 24 individuals, 71 alleles were found by cloning/sequencing and 76 were found by RSCA. Some of the MHC class II B alleles are found only by cloning/sequencing but not with RSCA and vice versa. There is also a tendency for RSCA to score more alleles than cloning (1-4 alleles are observed per individual via cloning and 1-6 via RSCA) but whether RSCA overestimates or cloning underestimates number of alleles is not yet clarified. There are several possible reasons for the observed discrepancies between RSCA and cloning.

The first reason to the discrepancies at the individual level may be that not enough clones were sequenced in the cloning. Alleles that are not amplified to the same extent as other alleles in the PCR due to non-optimal primer binding will fail to be found in cloning if not very many clones are sequenced. To test this, many more clones per individual than we used need to be picked and sequenced. In our lab, fosmid sequencing across MHC genes in one black grouse individual has uncovered two MHC class II loci (*BLB1 *and *BLB2*) (B. Wang *et al.*, in preparation), suggesting that a maximum of four MHC class II alleles can be present in black grouse. This is in line with the maximum of four alleles we observed in cloning. Our RSCA may thus overestimate number of alleles. However, at present we cannot rule out the possibility of copy number variations in MHC class II B in black grouse. Copy number variation in MHC has been observed in several species [18,19], including chicken where some individuals possess two BLB (*BLB1 *and *BLB2*) loci and other individuals three BLB loci [20]. *BLB1 *and *BLB2 *are possibly old duplications since they are positioned on either side of *TAPB *and are present also in turkey, which possesses a third BLB loci adjacent to *BLB2 *[20,54]. Copy number variation between one, two and three loci may explain the range of 1-6 RSCA alleles per individual. It is also possible that an individual can possess only one BLB allele in total even if black grouse possess between two and three BLB loci. It has been shown in other galliformes (red jungle fowl and pheasant) that identical MHC alleles can be present at different loci [36,55], so one allele is possible for a bird homozygous at two loci. We are attempting to elucidate a possible presence of copy number variation in an on-going MHC study.

The second reason for the observed discrepancies between RSCA and cloning/sequencing at the individual level may be non-optimal RSCA conditions. To minimize alternative folding structures RSCA run temperatures and hybridisation temperatures could have been tested for each FLR individually. Also, more FLRs could have been added to providing better resolution of alleles, but at the price of being time- and resource consuming.

Our result of the comparison between cloning/sequencing and RSCA is almost identical to a study of the domestic cat [21]. Using four FLRs we identified on average 2.96 alleles (range 1-4) via cloning and 3.17 alleles (range 1-6) via RSCA. In the feline study, using five FLRs, they found 3.0 alleles (range 1-4) by cloning and 3.9 (range 2-6) by RSCA. Copy number variation was found were some individuals carried two MHC class II DRB genes and some three, explaining the range from two to six DRB alleles per individuals (with RSCA). The allele range in black grouse could also be explained by similar copy number variation, as suggested above. Further, the feline study suggest that the deviations in allele number between the two methods could be the result of too strict rules for confirming an allele, (that were followed to disregard PCR artefacts) i. e. two independent PCR/cloning reactions. Paradoxically, another reason suggested for the deviations between the methods may be due to sequences that represent PCR artefacts. All this highlight the draw-backs of cloning and Sanger sequencing and the complexity of characterizing MHC variability.

The MHC study by Lenz et al. 2009 [28] comparing RSCA and cloning/sequencing found an even stronger congruence than we found between the methods. It is difficult to disentangle the reason for this since our study differs from Lenz et al. in many ways, for example in the selection of FLRs, the DNA quality, the run temperature and in the use of different electrophoresis instrument.

Since we cannot rule out that our RSCA genotyping protocol may overestimate the number of alleles, it may not yet be optimal for individual level studies where allele-specific correlations are sought for. Nevertheless, we believe that our RSCA protocol is ideal for fast and cheap population level studies of MHC diversity in black grouse and related species.

A future possibility is to multiplex the RSCA, using unique dyes for each FLR so that the different FLRs can be run in the same capillary, increasing by a factor three the speed of throughput. In this study, we used one black grouse FLR and three hazel grouse FLRs to successfully genotype black grouse MHC class II B. This leads us to believe that the protocol presented in this paper can directly work to genotype MHC class II B in other galliform species. At present, we are testing this hypothesis by performing RSCA with black grouse FLRs in hazel grouse and willow grouse, and preliminary results are indeed positive. We want to point out that for these newer studies we use non-denaturing matrix (for the MegaBACE™) from Genomac International (Prague) successfully. The RSCA can be adapted to other loci but MHC and should be of great use since it has the potential of separating any complex same-length sequences.

In a number of recent studies, genotyping of MHC is still performed solely by cloning and Sanger sequencing e.g. [56-58]. Cloning and sequencing of MHC alleles does not need optimization once the primers amplify the right region, however, we see two major drawbacks with this method. First, different types of artefacts can lead to false identification of true alleles, namely PCR artefacts (Taq errors, chimeras and heteroduplex formation in the last PCR cycle) and mismatch repair during cloning [26]. In RSCA, the number of artefacts is reduced since the PCR product is denatured before screening and thus heteroduplex formations are neutralized [26]. The second drawback with cloning and Sanger sequencing is that alleles at low frequency in the PCR can fail to be observed. The reason for this is when amplifying several MHC alleles simultaneously, all alleles may not be amplified to an equal concentration if the primers amplify certain alleles better than others. If this is the case, alleles can be missed in cloning, because many clones have to be picked to certify that all alleles are identified. In RSCA, the lower concentration alleles may have a better chance of being identified than in cloning, since only the height of the RSCA peak is lower than the other peaks if concentration is lower. In a recent study using RSCA, RSCA was in fact not the main genotyping method but were used to confirm MHC alleles obtained from sequencing [59].

When alleles from multiple loci co-amplify in the PCR, as the case for this black grouse study and many other studies, accurate genotyping can be very challenging. The reason for this is because gels and chromatograms become difficult to interpret since bands or peaks representing various alleles may overlap due to similar mobility. This is the case for PAGE-SSCP, CE-SSCP and DGGE. This problem is solved in RSCA since each sample is hybridised with several FLRs, so that alleles migrate differently with different FLRs, leading to a higher resolution. Recently, CE-SSCP has also been modified to discriminate alleles better than before by the use of overlapping PCR amplicons [60]. Other advantages in RSCA over PAGE-SSCP and DGGE are that the amount of toxic steps is greatly reduced as well as the labour since no handling of gels is needed. In theory, an advantage of both DGGE and PAGE-SSCP compared to RSCA, is the possibility of excising bands from the gels, reamplify and perform direct sequencing of new MHC alleles. For this to work, the bands have to be clearly separated in the gel. However, if sequences are needed in RSCA the procedure of classic cloning and sequencing can be greatly reduced by running RSCA on the clones. That is, samples that show new RSCA peaks can be cloned, and the clones can then be hybridised with an FLR and run through RSCA to identify the specific clone containing the new alleles. This procedure makes sequencing much cheaper since only clones including the new allele need to be sequenced. For further comparisons of the SSCP, DGGE and RSCA methods, see [27,28].

Next generation amplicon sequencing techniques has been increasingly used for MHC typing [29,61,62]. 454 amplicon sequencing, with individual tagging of PCR primers, seem to be a reasonable choice for highly variable multilocus MHC genotyping when other, more widely used methods, are not successful [27]. The major drawback with 454 amplicon sequencing seems to be the difficulties in distinguishing true alleles from artefact sequences, that exist in higher number than Sanger sequencing errors [29]. However, the large number of reads from pyrosequencing would allow for correction of typing errors [29].

In the context of population genetics, the purpose of MHC genotyping can roughly be divided into two different groups based on the level of precision. The first group may be referred to as "sequence level MHC genotyping" and the other group as "population level MHC genotyping". "Sequence level MHC genotyping" is necessary when MHC sequences from individuals are studied, to test for various forms of selection using patterns of nucleotide and gene diversity or to find allele specific mate choice or disease associations. On the other hand, "population level MHC genotyping" is ideal for non-model species studies when a simpler MHC genotyping method is being sought for. This might be the case in conservation genetic studies of adaptive genetic diversity, for example to identify populations vulnerable to changes in the environment. In "population level MHC genotyping" there is no per se need to know the details about nucleotide differences among the present alleles so no sequencing needs to be performed in the screening process.

We believe that RSCA has a great potential for "population level MHC genotyping" and is of good value even for "sequence level MHC genotyping". RFLP (Restriction Fragment Length Polymorphism) is a solid technique but involves gel preparations and radioactive work and is not as accurate as RSCA. RFLP cannot be used for "sequence level MHC genotyping" at a later stage. We suggest therefore that RSCA is a great first step in MHC studies in non-model species, where the level of detail and precision can be easily turned from "population level MHC genotyping" to "sequence level MHC genotyping" with the same method.

## Conclusions

In this study, we have developed a RSCA typing protocol for black grouse populations on a MegaBACE™ 1000 DNA Analysing System. We hybridised each PCR product with four different Fluorescently Labelled References in individual wells and separated them by electrophoresis. RSCA seems to be comparable at separating black grouse MHC class II B alleles as cloning and Sanger sequencing since we found 76 alleles by RSCA and 71 by cloning/sequencing among 24 individuals. There is however a tendency for RSCA to score more alleles than cloning at the individual level. Therefore, RSCA seems to be a promising method for studies when a simpler MHC genotyping method is being sought, for example, in conservation genetic studies for adaptive genetic diversity. Our study will also facilitate large scale MHC studies in species other than black grouse.

## Methods

### Study animal, samples and DNA extraction

#### The black grouse

Black grouse belong to the same order, Galliformes, and family, Phasianidae, as chicken [53]. The lekking black grouse is a species tied to taiga forest habitats. The females mate with only one male and the males do not provide any parental care [63]. In an earlier study [25] we showed that MHC class II B (BLB) and Y (YLB) are orthologous in chicken and black grouse and this finding was one of first examples of orthology in the avian MHC [9]. Black grouse and chicken possess approximately two to three BLB genes and two YLB genes. The BLB genes are more diverse and expressed than YLB genes [25]. 17 of the individuals used in this study were captured between 1989 and 2004 at study sites around Jyväskylä in central Finland (see [64] for a description of the study area). Black grouse were caught in winter flocks or when lekking and c. 1 ml blood samples were drawn from the brachial wing vein with a syringe using a heparinized needle. Red blood cells were separated from plasma by centrifuging at 12000 rpm for 5 min, and stored at 75% alcohol at +4°C until DNA-extraction. Older blood samples (before 1990) were kept frozen until DNA was isolated. Feathers from six other individuals (named "Ref" and "Ne36" in Table 2) were collected in North Pennines (England) and Netherlands. The moulted feathers were stored dry at room temperature for 1-20 months until DNA extraction. One sample (10222) was sampled in Norway and the tissue type is muscle. For blood, genomic DNA was extracted mainly using a standard phenol-chloroform procedure [65], but salt extraction [66] was also used. Genomic DNA from feathers and muscle was extracted using DNeasy Tissue kit as described in [67]. Birds that were caught, hunted or released were done so under the permission, legislation and guidance of the relevant authorities of the country of origin.

#### Hybridisation

PCR were performed as described under the section "Construction of RSCA library". For production of FLRs, see the section "FLR amplification" in the result part. For individuals, 3.0 µl of PCR product was mixed with 2.0 µl of FLR and for clones 2.0 µl of PCR product was mixed with 2.0 µl FLR. The ratio between FLR and PCR product was optimised for each FLR, so FLRs were diluted individually. The hybridisation was performed using a PCR thermo cycler starting with denaturation at 95°C for 10 min, and then cooling by 1°C/s until 55, then heteroduplex formed for 15 min at 55°C until a cooling step at 4°C for 15 min. The heteroduplex product was then stored at 4°C.

#### Capillary array electrophoresis

The hybridisation product was mixed with 4.0 µl cold ddH_2_O. 2.0 µl of the diluted heteroduplex product was then mixed with 0.45 µl ET400R-Size standard (GE Healthcare) and 7.55 µl ddH_2_O. The capillary array electrophoresis was performed on MegaBACE™ 1000 DNA Analysing System (GE Healthcare). First, samples were preinjected to remove excess salt and next, the capillaries were rinsed. Thereafter, capillaries were filled with 3% non-denaturing Long-Read-Matrix (polymer) (GE Healthcare) under high pressure. Lastly, 96 samples were injected at 3 kV for 45 seconds and run for 60 min at 30°C and 10 kV. During the typing period, the capillaries were used either for sequencing or microsatellite genotyping when not used for RSCA, so we alternated between non-denaturing matrix and denaturing matrix.

### Data analysis

MegaBACE fragment profiler™ version 1.2 was used to analyse the RSCA peaks. CodonCode Aligner version 2.06 was used to identify the new sequences. The difference in number of alleles in RSCA versus cloning and sequencing were tested in a paired Wilcoxon signed rank test in R v2.12.0.

## Competing interests

The authors declare that they have no competing interests.

## Authors' contributions

TMS designed the study, performed the run of the experiments and wrote the manuscript. JH contributed funding, supervised experiments and participated in writing the manuscript. All authors read and approved the final manuscript.

## Supplementary Material

Additional file 1 **RSCA library**. Table of the RSCA library showing RSCA migration values for each of 16 MHC class II B alleles hybridised separately with Fluorescently Labelled References FLR-Tete1, FLR-Bobo1, FLR-Bobo2 and FLR-Bobo3. Click here for file
