# Supplementary material for: Genotyping of black grouse MHC class II B using reference Strand-Mediated Conformational Analysis (RSCA)
Source: BMC Res Notes. 2011 Jun 14;4:183. doi: 10.1186/1756-0500-4-183 (PMC3141517; doi:10.1186/1756-0500-4-183)
Supplement: Additional file 1 — RSCA library. Table of the RSCA library showing RSCA migration values for each of 16 MHC class II B alleles hybridised separately with Fluorescently Labelled References FLR-Tete1, FLR-Bobo1, FLR-Bobo2 and FLR-Bobo3. [file 1756-0500-4-183-S1.DOC]

**Additional file 1: RSCA library**

Table of the RSCA library showing RSCA migration values for each of 16 MHC class II B alleles hybridized separately with Fluorescently Labelled References FLR-Tete1, FLR-Bobo1, FLR-Bobo2 and FLR-Bobo3. One column pair of “Min” and “Max” refers to one peak/allele were “Min” is the minimum observed migration value in the electropherogram and “Max” is the maximum observed migration value. The “Min” and “Max” can thus be read as the migration value ranges for each allele. Using FLR-Bobo2, one, two or three peaks have been observed for each allele which explains three “Min” and “Max” column pairs. In FLR-Bobo3, two peaks were observed in one of the alleles.

|  | **FLR-Tete1** |  | **FLR-Bobo1** | | **FLR-Bobo2** | |  |  |  |  | **FLR-Bobo3** | |  |  |
| --- | --- | --- | --- | --- | --- | --- | --- | --- | --- | --- | --- | --- | --- | --- |
| ***Tete BLB*** | Min | Max | Min | Max | Min | Max | Min | Max | Min | Max | Min | Max | Min | Max |
| ***1*** |  |  | 199.8 | 209.8 | 169.7 | 173.7 |  |  |  |  | 220.6 | 232.6 | 193.0 | 199.0 |
| ***2*** | 199.0 | 203.0 | 164.0 | 172.0 | 172.6 | 176.6 | 180.0 | 186.0 |  |  | 209.3 | 215.3 |  |  |
| ***3*** | 171.5 | 175.5 | 204.0 | 206.0 | 170.0 | 174.0 |  |  |  |  | 225.0 | 231.0 |  |  |
| ***4*** | 171.5 | 175.5 | 184.0 | 190.0 | 166.0 | 172.0 |  |  |  |  | 228.1 | 238.1 |  |  |
| ***5*** | 204.4 | 216.4 | 190.4 | 196.4 | 210.7 | 222.7 | 205.0 | 209.0 |  |  | 183.1 | 191.1 |  |  |
| ***6*** | 202.9 | 208.9 | 184.6 | 188.6 | 198.4 | 202.4 | 213.4 | 217.4 | 201.9 | 204.9 | 222.9 | 228.9 |  |  |
| ***7*** | 181.9 | 185.9 | 169.7 | 173.7 | 179.3 | 183.3 |  |  |  |  | 201.7 | 205.7 |  |  |
| ***9*** | 179.6 | 187.6 | 166.4 | 170.4 | 175.9 | 181.9 |  |  |  |  | 207.4 | 211.4 |  |  |
| ***11*** | 166.3 | 170.3 | 186.1 | 194.1 | 173.8 | 177.8 |  |  |  |  | 224.6 | 230.6 |  |  |
| ***13*** | 174.4 | 178.4 | 175.1 | 179.1 | 180.2 | 184.2 | 188.7 | 192.7 |  |  | 215.2 | 219.2 |  |  |
| ***14*** | 175.1 | 179.1 | 176.4 | 180.4 | 179.8 | 183.8 | 187.9 | 191.9 |  |  | 214.0 | 218.0 |  |  |
| ***16*** | 175.2 | 181.2 | 169.0 | 173.0 | 178.5 | 182.5 |  |  |  |  | 205.0 | 209.0 |  |  |
| ***18*** | 172.0 | 176.0 | 184.7 | 188.7 | 169.4 | 173.4 | 205.0 | 211.0 |  |  | 198.0 | 204.0 |  |  |
| ***21*** | 199.0 | 203.0 | 192.0 | 198.0 | 203.0 | 207.0 |  |  |  |  | 213.0 | 217.0 |  |  |
| ***22*** | 183.0 | 185.0 | 204.0 | 208.0 | 178.0 | 182.0 |  |  |  |  | 204.4 | 212.4 |  |  |
| ***23*** | 166.0 | 170.0 | 187.4 | 193.4 | 172.6 | 176.6 |  |  |  |  | 223.7 | 229.7 |  |  |
